# Supplementary material for: A Scoping Review of the Health Impact of the COVID-19 Pandemic on Persons Experiencing Homelessness in North America and Europe
Source: Int J Environ Res Public Health. 2022 Mar 9;19(6):3219. doi: 10.3390/ijerph19063219 (PMC8954292; doi:10.3390/ijerph19063219)
Supplement: Supplementary file 1 [file ijerph-19-03219-s001.zip › ijerph-1605301-supplementary.pdf]

Supplementary Table S1: Detailed data Extraction.

Title	Author(s), (year), country	Study Design	Impact on persons experiencing homelessness (PEH) relevant to review
A real-world ten-week follow-up of the COVID outbreak in an outpatient drug clinic in Salamanca (Spain)	Aguilar et al. (2021), Spain	Longitudinal ecological study	Study conducted with social distancing and facemasks. Hospital detoxification and inpatient dual disorder units closed (16 March 2020). Addiction and dual disorders unit developed telehealth service, two new healthcare programmes (mental health and one for PEH only). Access to psychiatrists, psychologists, nurses, social workers, and nursing assistants, pharmacological, psychological/injection treatments. Increases in number of relapses and psychological destabilisation. Staff/social workers helped clients find housing, manage admission into therapeutic communities and maintain contact with psychosocial support team.
COVID-19: opportunity to improve crisis responses to homelessness?	Aitken et al. (2021), UK	Case series	*Individual A:* Referred to hospital in-reach team by ward staff. Previously living in camper van and socially isolated. First time availing of homeless services. Referred to private renting service, found new accommodation, reconnected with family within 2 weeks. Process expedited due to pandemic.<br>*Individual P:* History of untreated alcohol use disorder (AUD), trauma, post-traumatic stress disorder (PTSD). After being offered place in hotel, P: stopped drinking, engaged with supports, planned for the future, ate well and improved physical health. Offered supported accommodation in hostel, improved his behaviour. P became calmer, more stable, comfortable after knowing he could stay in hotel all day and when mealtimes were, his worries reduced, supports approached him, he did not have to retell his story, could rely on communication between support services.<br>*Individual W:* Experiencing homelessness for 9 years, reported fear of statutory services. Sleeping rough upon admission to Emergency Department. Withdrawing from heroin, wanted to self-discharge, started on methadone and stayed in hospital extra night due to pandemic. Hospital in-reach team visited, built rapport, supported daily with housing options. Immediately given homeless priority status and Housing Officer. Provided supported accommodation at discharge. One year later is drug free, no hospital admissions and lives in supported accommodation, is engaged in 12-step drug program, volunteers and reconnected with family. Improved health receives regular support/food/access to methadone.<br>*Individual M:* Lived rough on street >10 years. Received food, money, clothing from public pre-pandemic. During pandemic: met new professionals, engaged with health services, social work, other street-based services, benefits for the first time. Moved into accommodation after learning about coronavirus disease (COVID-19) isolation measures. M remains in accommodation, has support staff on-site, improved physical health after accessing food and support systems, started reconnecting with family.

			Milestone House Intermediary Care Unit (MHICU): places to recover/isolate, identified PEH avoiding temporary accommodation, eased hospital flow, managed comorbidities, assessed and addressed chronic health/social/housing needs. Average stay: 25 days. Planned discharge to suitable accommodation (56%), discharged unplanned (28%), ongoing (16%). Primary Care engagement: re-engaged (32%), registered with general practitioner (GP) (18%), community physiotherapy (10%), cognitive assessment for housing (2%), social work and substance use services (2%). Hepatitis C virus (HCV) treatment: commenced (6%), re-engaged (8%), supported to completion (4%). Hospital outpatient management: supported with outpatient appointments (32%), chronic health treatment (18%). Hospital admission prevention: MHICU prevented acute hospital attendance (12%), and reduced time in hospital (18%). Housing: moved from no fixed abode to appropriate accommodation (18%), moved to more appropriate accommodation (14%), returned to altered tenancy (2%). Community support: re-engaged (20%), newly referred to addiction services (8%), housing support (30%), homecare support (4%).<br>*Welcome Hub:* newly developed during COVID-19, alternative to Night Shelter/congregated settings, admitted 700 within 6 months of opening, roughly 50% newly accessing homeless services, provided 24-hour support, bedroom, changing/washing facilities. This improved dignity and willingness for PEH to engage with services. Original Night Shelter ceased as a congregate model and opened as Welcome Hub.
"Locked down outside": Perception of hazard and health resources in COVID-19 epidemic context among homeless people	Allaria et al. (2021), France	Mixed methods	Serological tests for severe acute respiratory syndrome coronavirus 2 (SARS-CoV-2) offered: 3% positive. 67% had health insurance (16% living rough, 41% in emergency shelters, 43% in squats); 42% changed accommodations during COVID-19 (66% living rough, 41% in emergency shelters, 38% in squats); 56% actively consuming tobacco; 25% actively consuming alcohol; 48% had at least one comorbidity; 23% had psychiatric or substance use disorder. 17% had unmet physical healthcare needs: (17% living rough, 31% in emergency shelters, 12% in squats); 24% had unmet mental health needs (33% living rough, 28% in emergency shelters, 17% in squats). 50% of those with psychiatric conditions reported unmet psychiatric needs. Reported compliance with: wearing a mask (72%), hand washing (75%), social distancing (75%), overall compliance (80%). Difficulties accessing: water (29%), food (48%), hygiene products (35%), cleaning products (31%). PEH in emergency shelters had highest overall self-compliance rates with COVID-19 prevention strategies (vs. PEH in streets or slums); compliance higher among PEH in squats/slums (vs. streets). PEH in shelters had at least twice the proportion reporting a psychiatric or substance use disorder and more likely to report previous COVID-19 infection (vs. streets or slums/squats). PEH in slums reported lower unmet mental health needs (vs. emergency shelter). PEH sleeping on streets more likely to report difficulties accessing resources (vs. emergency shelters) for: food (60% vs. 24%), water (39% vs. 5%), hygiene (49% vs. 8%), cleaning supplies (36% vs. 5%).<br>Some shelters overcrowded, some reduced crowding, offered shielding. Cleaning practices followed.<br>PEH feared catching virus (difficulties social distancing). Those living rough or in squats: daily struggles

			accessing water, social distancing and maintaining relationships. Some practised social distancing and reduced sharing equipment/items. Those in slums had access to water, toilets. Some sleeping rough had access to toothbrush, shaving equipment. Some PEH feared COVID-19 but no more than pre-exiting health concerns. Some lost accommodation (owners feared COVID-19). Some left family/friends to move between public spaces and emergency shelters, left institutions (e.g. prison), newly PEH.<br>-*Difficulties accessing essential resources:* Support from public and local organisations, and access to food, hygiene reduced. Better knowledge of food services was associated with better access. Mobile food distributors provided food. Residents/public redistributed the food packages. Families in slums received food vouchers, allowing them to buy food, female and baby hygiene supplies.
Drug Overdose Deaths Before and After Shelter-in-Place Orders During the COVID-19 Pandemic in San Francisco	Appa et al. (2021), United States	Cross-sectional	Significant differences between PEH overdose death rates (23 vs. 34%) before the pandemic commenced (1 July 2019-16 March 2020) and during pandemic respectively (17 March-30 November 2020).
Implementation of Rapid and Frequent SARS-CoV2 Antigen Testing and Response in Congregate Homeless Shelters	Aranda-Díaz et al. (2021), United States	Pilot study	Monthly reverse real-time polymerase chain reaction (rRT-PCR) testing across 12 shelters conducted for pilot programme. Positive individuals referred to isolation and quarantine (I&Q) for case investigation/contact tracing. Symptomatic participants isolated. Confirmed negative participants returned to shelter.<br>58% remained in shelter for duration of study. 48% of eligible residents tested at least once. 24% tested at each event. 53% on-site tested at each event. 37% discharged/admitted. 5% tested positive via rRT-PCR (all asymptomatic). 3% tested positive via BINAXNow (10% symptomatic). 80% transferred to I&Q that day; Of those transferred to I&Q, 38% confirmed positive, 25% did not receive confirmatory test, 38% tested negative (confirmatory test). 40% of shelters had positive cases. 30% of shelters had outbreaks. Shelters with outbreaks were often large, had transient resident populations.
Addressing COVID-19 Among People Experiencing Homelessness: Description, Adaptation, and Early Findings of a Multiagency Response in Boston	Baggett et al. (2020), United States	Case study	COVID-19 citywide care model developed: portable toilets, personal protective equipment (PPE), beds, medical staff, front-door screening, isolation/management venues, exposure screening, contact tracing, quarantine, shelter-based infection control, real-time surveillance. Guests with cough/shortness of breath referred to pop-up COVID-19 testing sites. 2 tents (symptomatic patients and quarantine COVID-exposed asymptomatic) designated for isolation. Positive polymerase chain reaction (PCR) test results were hospitalized/transferred to PEH COVID-19 care unit.<br>Area shelters deployed disinfection, environmental/administrative controls, decongestion strategies. Surveillance strategy identified PEH with COVID-19/symptoms requiring alternative care plans/follow-up. COVID-19 care unit expanded. More beds available from reopened facilities. City/state funding

			constructed field hospital for PEH/marginally housed. Addiction/medical/psychiatric treatment shifted to telehealth. 33% COVID-19 positive.
Prevalence of SARS-CoV-2 Infection in Residents of a Large Homeless Shelter in Boston	Baggett et al. (2020), United States	Cross-sectional	Collaboration between healthcare for PEH programme, city/state public health agencies and community providers developed COVID-19 response strategies (respiratory symptom screening at shelter front doors, sooner referrals for testing/isolation of those with symptoms, appropriate treatment for those with positive test results, contact tracing of positive results).<br>Symptom prevalence (Total%; COVID-19 positive%; COVID-19 negative%): body temperature >37.8°C (1%; 1%; 1%), any symptoms (12%; 12%; 11%), cough (8%; 8%; 8%), shortness of breath (1%; 1%; 0.4%), nasal/sinus (2%; 1%; 2%), diarrhoea (1%; 1%; 1%), subjective fever/chills (1%; 2%; 0.4 %), miscellaneous (2%; 2%; 2%).
The impact of Covid-19 on homeless service providers & homeless people: The migrant perspective	Barbu et al. (2021), Poland; England; Germany; Spain; Denmark	Report	Some migrants experience homelessness feared touching money, close contact with others. Lack of information-in Poland, some unaware why shelters closed, less people on the streets. Sheltered PEH kept for isolation found their situation difficult, frustrating, hard to accept, increased nerves and conflicts. PEH with AUD found their cravings for alcohol increased with the added measures for confinement and isolation. Unstable finances following public health guidance difficult. Germany-limited access to food; Spain-loss of social connections, access to shower, food, drink and pocket money; England-lack of information/advice contributed to sense of loneliness; Denmark-mental health deteriorated, public spaces and water taps closed, no place to stay, socially distance or shield from the virus. Toilets later re-opened, and water sources provided. Some accessed hand sanitisers, or unable to follow recommended guidelines due to lack of safe residency.<br>Case studies: Some lost accommodation due to inability to find jobs, access welfare payments, using rent money for food. Support provided for alcohol use after referral to hospital. Poor mental health, limited food supply due to food aid service closures. Some food support provided.
Implementation of a Recuperation Unit and Hospitalization Rates Among People Experiencing Homelessness With COVID-19	Barocas et al. (2021), United States	Interrupted time series	PEH admitted to: medical centre (13%), and COVID-19 Recuperation Unit (CRU; 84%). 28% reduction in hospitalisations for COVID-19 positive PEH.
Loneliness among Homeless Individuals during the First Wave	Bertram et al. (2021), Germany	Cross-sectional	Frequency of sharing sleeping space with >3 people (range: 1=never, 4=always): *M*=2.9. Absence of chronic alcohol consumption (64%), presence of chronic alcohol consumption (36%). Self-perceived risk of contracting virus (range: 1=very low, 5=high): *M*=1.8. 49% met cut-off point for loneliness.

of the COVID-19 Pandemic			Predictors of loneliness: being single, high frequency of sharing sleeping space with more than three people, high self-perceived risk of contracting COVID-19. Predictors of decreased feelings of loneliness: being female, from neighbouring country of origin. Association between country of origin and loneliness was lost after addressing missing data in analysis.
COVID-19: The forgotten cases of hidden exiles.	Bihan et al. (2021), France	Retrospective	All completed SARS-CoV-2 tests. Positive cases monitored in medical homeless facility or hospitalised. Those testing negative with comorbidities were sheltered in hotels. *Squat A:* no showers, one water source, toilets. By study completion, more showers and toilets put in. Social distancing and containment not possible. Squat A was dismantled (31 August 2020). *Squat B:* had showers, toilets, running water. By study completion date, more showers, masks, hand sanitiser and toilets were added. Social distancing/containment was possible. Positive cases isolated in single rooms. *Squat A outcomes:* COVID-19 positive (38%), 4% had cough (4%; 50% of these were positive cases), no hospitalisations. Of positive cases, 16% transferred to the COVID-19 homeless care centre and 5% did not physically receive their test result. *Squat B outcomes*: COVID-19 positive (3%), had cough (5%; all negative cases), no hospitalisations/transfers to COVID-19 homeless care centre. For both squats: approximately 1% were sharing rooms.
Enhanced Telehealth Case Management Plus Emergency Financial Assistance for Homeless-Experienced People Living With HIV During the COVID-19 Pandemic	Brody et al. (2021), United States	Retrospective cross-sectional	Those with phones informed about COVID-19 prevention/access to medical treatment. Those without (w/o) phones provided mobiles with unlimited minutes for staff contact. Facemasks and hand sanitizer provided by mail/at walk-in clinics. All contacted biweekly re: patients access to COVID-19 supplies, medical needs/prescriptions updates, other COVID-19 risk factors (food security, housing stability, bills, telephone access, and transportation needs). Food gift cards/deliveries, utilities and rental support, vouchers for short-term hotel/motel stays, transportation to urgent medical visits provided. Currently unhoused patients kept: 4.95 case management contacts on average, 57% of human immunodeficiency virus (HIV) appointments. 33% tested for COVID-19: 34%=positive.
Sheltering the Homeless during COVID-19 in San Jose, California	Brown et al. (2021), United States	Case study	Shelter protocols implemented: temperature/symptom checks, sanitising regularly, hand sanitizer/PPE available, social distancing enforced, designated location for isolation of positive cases. Glove and masks enforced for children playing video games. Two counties provided: additional emergency shelters, shower/laundry trucks. Food and necessities were distributed to PEH in motels, hotels, and temporary shelters. Unsheltered PEH had service-managed shelter or quarantine options, hygiene services delivered, and health support. 86% shelters increased number of residents or beds between April-July, 2020. 45% of residents tested for SARS-CoV-2: .01% positive.
Prevalence of COVID 19 Positive Cases Presenting to a	Cardenas et al. (2021),	Cross-sectional	All tested for COVID-19. Vital signs; presence of: cough, fever, shortness of breath, diarrhoea; whether individual is presenting from congregate living arrangement assessed at admission. Symptomatic/people living in congregate environments sent to isolation. Asymptomatic patients awaiting results went to

Psychiatric Emergency Room	United States	retrospective chart review	individual isolation rooms, integrated with rest of population in receipt of negative result. Facemasks and social distancing encouraged. Positive patients stayed in isolation rooms.<br>Positive cases (57%=PEH). PEH positive for: amphetamines (54%), cannabis (46%), cocaine (8%). 38% tested negative for substances. Diagnoses: mood (8%), psychosis (77%), substance use disorder (SUD; 46%), dual diagnosis of psychosis and SUD (31%); 15% had at least one comorbidity: ulcerative colitis (8%), diabetes/hypertension (8%); 8% reported a symptom (cough).
Morbidity and Mortality Among Adults Experiencing Homelessness Hospitalized With COVID-19	Cha et al. (2021), United States	Cross-sectional	Smoking: currently (46%), formerly (21%), never (33%). Alcohol abuse: Currently (34%), formerly (10%), never (56%). Substance abuse/dependence (3%), mental health diagnosis (8%), underlying health condition (83%), hypertension (44%), obesity (24%), diabetes (16%), chronic lung disease (22%), chronic metabolic disease (19%), cardiovascular disease (25%), gastrointestinal/liver disease (19%), neurologic disorder (24%), immunosuppressive condition (2%), renal disease (5%), blood disorder/haemoglobinopathy (5%), rheumatalogic/autoimmune disease (1%), wheelchair dependent (6%).<br>COVID-19 admission symptoms: cough (54%), fever/chills (53%), shortness of breath (51%), chest pain, headache, nausea/vomiting, myalgias (20-23%). Median length of hospitalisation=four days, invasive mechanical ventilation (11%)-most commonly used for over 65 years old and those with no underlying health concerns. Supports: vasopressor (6%), systemic steroids (16%), renal replacement therapy/dialysis (4%). 1% died (all symptomatic, mostly male, had underlying health conditions, aged ≥50 years).
HIV and Hepatitis C Linkage-to-Care Initiative for New Orleans Residents Experiencing Homelessness During the COVID-19 Pandemic	Cironi et al. (2021), United States	Non-concurrent cohort	Four temporary housing shelters created to minimise COVID-19 spread/provide housing. Access to: case managers. Some left shelters voluntarily when placed in long-term housing. HCV/HIV/COVID-19 screening, counselling, linkage-to-care initiative implemented in all temporary shelters. PEH provided pre-exposure prophylaxis (PrEP) medication education. Seropositive cases referred to healthcare clinic for follow-up with primary care provider. Healthcare clinic organised transportation to any healthcare visits. Department of housing provided: in-house case worker (assist clients to move through treatment processes)-link positive patients to healthcare clinic, organised private telehealth appointments. Some accessed phones: others used their case workers computer for telehealth.<br>Characteristics: history of intravenous (IV) drug use (25%)/sharing injection equipment (8%), HCV seropositive (25%), HIV seropositive (3%), HCV/HIV co-infected (1%). Associated with HCV seropositivity: history of IV drug use and sharing injection equipment and those older than 40 years old.<br>HCV treatment cascade: >60% HCV seropositive patients unaware of HCV infection pre-test. HCV seropositive and never received treatment previously: 100% informed of results, 84% linked to healthcare clinic (follow-up), 60% attended appointment with healthcare clinic provider, 52% used transport for confirmatory start of care labs, 40% collected medication to commence treatment, 8% cured. Only PEH chronically infected with HCV eligible for medication (92%). 28% of HCV seropositive PEH with no previous cure, needed case manager to connect with clinic for treatment. 86% of these did not continue

			after phone-call with healthcare clinic treatment manager. Of those completing pre-treatment laboratories: 38% needed clinic-provided transportation. 80% of HCV-seropositive patients had insurance. 10% of those on active treatment did not have insurance with clinic. 0% of HCV-seropositive PEH actively taking PrEP; 28% never heard of PrEP medication previously.
Food insecurity in times of Covid-19 -an insight into a deepening crisis	Dempsey et al. (2021), UK	Report	Free food parcels delivered/provided in collaboration with care homes. Barriers to food before pandemic: financial strains, lack of support, irregular incomes. During pandemic: PEH chose between food and other basic needs. Community kitchen helped build: positive relations, supportive environment, new skill sets (e.g. maintaining healthy diet strategies), friendships, greater sense of dignity (compared to food parcels being delivered). COVID-19 shifted kitchens holistic approach to mass food delivery: support networks lost, relationships broke down/lost contact with PEH. Recovery, mental health, self-esteem, dignity was exacerbated by these changes. COVID-19 ceased food aid services: isolation and lack of socialising among PEH increased (some lost food deliveries-ineligible w/o official shielding advice). Travel restrictions and buying locally significantly reduced choice of shops/supplies.
SARS-CoV-2 antibody prevalence among homeless people, sex workers and shelter workers in Denmark: a nationwide cross-sectional study	Eriksen et al. (2021), Denmark	Nationwide cross-sectional seroprevalence	76% experiencing homelessness. 7% seropositive, 45% consumed drugs, 49% consumed alcohol, 12% engaged in sex work, 59% previously tested (COVID-19). Current smokers (79%): tobacco (52%), cannabinoids (29%), cocaine (4%), heroin (3%). Of seropositive PEH, 40% had symptoms. PEH engaging in sex work (11% seropositive) more likely to be female or seropositive and less likely to smoke or use drugs intravenously compared to PEH not engaging in sex work. 4% of PEH did not follow recommended COVID-19 prevention guidelines. PEH more likely to be older, current smoker compared to staff. PEH less likely to use alcohol and previously tested compared to staff. Seropositive PEH more likely to consume heroin; seronegative PEH more likely to consume cocaine. PEH with: at least one symptom (40%); three or more symptoms (23%).
Virtual care expansion in the Veterans Health Administration during the COVID-19 pandemic: clinical services and patient characteristics associated with utilization	Ferguson et al. (2020), United States	Longitudinal	4% of veterans experiencing homelessness.<br>Use of virtual care: never (1%), existing (6%), new (1%). Use of video care: never (4%), existing (7%), new (7%). PEH more likely to use virtual care before and/or after COVID-19, and increased for video care. New users of video care more likely to be PEH (7% vs 4%). Veteran PEH were 11% less likely to use video care compared to non-homeless counterparts during pandemic. Odds of using: virtual care higher among urban PEH; video care higher among rural PEH.

Assessment of contact tracing for COVID-19 among people experiencing homelessness, Salt Lake County Health Department, March-May 2020.	Fields et al. (2021), United States	Cross-sectional	COVID-19 testing via real-time polymerase chain reaction (RT-PCR). Contact tracing involved: providing quarantine/isolation recommendations, distributing resources on I&Q housing/medical care. Majority of positive PEH cases derived from outbreak in men's shelter. COVID-19 testing freely available.<br>84% positive cases from one shelter (rest from 7 additional shelters). Comorbidities: yes (7%), no (12%), unknown (81%). Smoking status: current (6%), former (0%), never (12%), unknown (82%). Symptoms: yes (39%), no (37%), unknown (25%). Of those reporting symptoms: cough (60%), shortness of breath (35%), muscle aches (29%), chills (26%), subjective fever (25%), runny nose (25%), sore throat (22%), headache (20%), nausea/vomiting (20%), diarrhoea (15%), fever > 38°C (14%), loss of taste (14%), loss of smell (12%), abdominal pain (8%), other (32%). Hospitalised: yes (14%), no (86%), unknown (1%), symptomatic (29%). Died: yes (1%), no (99%), symptomatic (3%). PEH more likely to be hospitalised. Contact tracing results: interviewed (55%), not interviewed-refused (2%), not interviewed-unable to locate/contact (43%); lost to follow-up (14%); provided contacts (12%), symptomatic, reported contacts (19%), asymptomatic, reported contacts (10%); contact=family/household member (32%), contact=experiencing homelessness (38%); contacts tested: yes (62%), no (18%), unknown (20%); positive contact cases (16%).<br>General population contacts were more likely to be interviewed, nearly twice as likely to provide contacts, family members as contacts, have contacts tested, more likely for contacts to have symptoms, provided roughly 16-times more contacts. Contacts of PEH were more likely to be lost at follow-up, provide incomplete/inaccurate contact information, or experiencing homelessness.
Self-reported impacts of the COVID-19 pandemic for people experiencing homelessness in Sacramento, California	Finnigan et al. (2021), United States	Cross-sectional	The county, local service providers and state government collaborated to attain rented hotel rooms and other quarantine/isolation spaces (8 April-9 September 2020). California placed shelter-in-place order on 19 March, 2020.<br>Loaves & Fishes (L&F) homeless shelter survey: 78% unsheltered, 74% experiencing homelessness for at least one year, 53% had a physical/mental disability, 46% had a psychiatric mental health condition, 100% received food from community organization, 42% received benefits for food, 6% had access to work for pay. L&F provides: meals, sanitation services, clothing, medical services, and a school program for children experiencing homelessness. Work for pay was lower in L&F participants compared to low-income household Californians. There were more self-reported economic impacts than disease impacts recorded.<br>Self-reported impacts of pandemic: believe they had (3%) or were exposed to COVID-19 (11%), tested for COVID-19 (61%), avoided shelters due to fear of COVID-19 (27%), income was lower in October 2020 than February 2020 (33%), income remained about the same (62%), laid off/lost job due to pandemic (16%), received stimulus check from government (45%), monthly income of $500 or less (67%), no income (32%), some PEH income never changed because they "never really had any anyway". Some avoided shelters for reasons other than fear of virus (e.g. too crowded, lack of privacy, risk of COVID-19 was preferred to

			remaining unsheltered). People with monthly incomes over $500 were more likely to receive stimulus check compared to those with monthly incomes below (70% vs. 31%).
Homelessness Monitor England 2020: COVID-19 Crisis Response Briefing	Fitzpatrick et al. (2020), UK	Longitudinal	Clear instructions, extra funding, relaxing distribution of benefit payments and communication between local and central governments made it easier to shelter PEH. PEH moved from shelter to hotel in four days. " Everyone In" programme identified PEH and kept people off the streets. Everyone In programme: commercial hotel rooms allocated, toilet, bath, supports and three meals per day. However, this programme not possible outside urban cities-less opportunity to re-purpose hotels. Everyone In used test-triage-cohort approach. Previously unidentified PEH newly approaching services (could not stay with friends/family or continue working in exploitative jobs). Those coming from streets were typically asymptomatic. Those using drugs/alcohol may be more suited to housing first (no substance supports in hotels). Early challenges: evictions/conflicts; getting long-term PEH to return to hotels after being on streets/socialising with familiar social circles. Mostly newly PEH entering shelters. Police supported local services to locate PEH.<br>Concerns for what may happen to PEH (20% ineligible for housing benefits) when Everyone In programme ceases/PEH back on streets. Accessing private rented sector was easier than before pandemic. Some saw improvements in availability/supports for domestic abuse. Some PEH had no access to adult social care. PEH commenced taking medication during the pandemic (lack of financial support from streets).
Assessment of a Hotel-Based COVID-19 Isolation and Quarantine Strategy for Persons Experiencing Homelessness	Fuchs et al. (2021), United States	Retrospective cohort study	Five I&Q hotels: delivered medical/behavioural health services; team of nurses, health workers, and security staff available; provided on-site nurse monitoring of client symptoms; bi-daily phone call wellness checks; meals adhering to dietary restrictions; hygiene kits; addiction medicine physician teleconsultation; pharmacology for opioid use disorder (OUD), counselling for SUD; harm-reduction strategies; access to safe consumption supplies, designated smoking areas, intranasal naloxone, nicotine replacement, medical cannabis, and managed alcohol programme; alcohol dosed bi-daily; other services (laundry services, bi-daily phone calls from I&Q counsellors). Median hotel stay=10 days. Referred from hospital (36%), sheltered/unsheltered (50%). 46% lab confirmed COVID-19, 38% awaiting results, 15% close contacts. 95% for retention and voluntary premature discontinuation of I&Q: 81% completed I&Q stay.<br>Multivariate regression predictors-premature discontinuation of I&Q: close contacts, unsheltered, <40 years old, Black/African American identity, referred to study later. Sensitivity analysis predictors of premature discontinuation: younger age, female, unsheltered, close contact, referred to study later.<br>Hospital transfers to I&Q: successful transfers (63%), referred once (56%). 2+ referrals (3%): 76% completed I&Q (62% lab confirmed COVID-19); 4% retested for COVID-19 (69% hospitalised for other concerns), no deaths. 21% ineligible for I&Q (42%-mental health/substance use needs beyond resources). More successful I&Q hotel transfers than overall COVID-19 admission to hospital during study period. Mean inpatient stay=3.9 days.

Case 21-2020: A 66-Year-Old Homeless Man with Covid-19	Gaeta et al. (2020), United States	Case report	Male PEH characteristics: Presented for evaluation (dry cough and rhinorrhoea) that morning. Rest, oral hydration, ibuprofen, cough suppressants recommended. Client returned to congregate shelter. 7 days later-patient tested, showed no new symptoms. 2 days later-positive for COVID-19, transferred to newly developed COVID-19 medical isolation ward. Reported cough, nasal congestion, fatigue, headache, and sore throat at admission. Over next 5 days-fever, non-productive cough maintained and malaise/anorexia developed. Did not receive supplemental oxygen; faint bilateral wheezing; encouraged to leave bed, walk around.<br>COVID-19 unit: areas with or w/o exposure to virus, separate areas for putting on/removing PPE. Creation of alternative care sites for I&Q. Two tents created near local homeless shelter: 1) isolation site for symptomatic persons awaiting results (access to own bathroom), 2) quarantine site for asymptomatic persons with known/suspected exposure to virus (vital-sign monitoring station, food, facemasks available). Performed: front door screening; testing symptomatic/contact investigating persons; two-item screening tool (cough, shortness of breath), if either present-temperature taken, and referred to pop-up testing site. Convention and Exhibition centre generated 500 beds. Homeless shelters allowed use university accommodation nearby to limit crowding. Follow-up care when discharged from discharge planners.
Risk Factors for Severe Acute Respiratory Syndrome Coronavirus 2 Infection in Homeless Shelters in Chicago, Illinois-March-May, 2020	Ghinai et al. (2020), United States	Point prevalence	Testing offered to all. Shelters with point prevalence ≥5% offered further testing 1–2 weeks later. Most shelters regularly cleaned environment, enforced facemask wearing, practised hand hygiene, symptom screening, isolated symptomatic cases.<br>41% of PEH tested more than once: 30% positive. Sleeping arrangement: single room (18%); share room with 2-4 people (12%), 5-8 people (10%), 9-20 people (12%), >twenty people (47%). Smoking status: never (38%), current (42%), former (20%). Could leave and return to shelter during day (49%). Of COVID-19 positive PEH: 27% reported symptoms (vs. 16% w/o COVID-19 diagnosis), 13% hospitalised (33% of these required admission to intensive care unit; ICU), <1% died. PEH COVID-19 symptoms: cough (63%), congestion/runny nose (43%), subjective/measured fever (39%).<br>Shelters characteristics: mean occupancy=65, number of residents per 1000 square feet=4, reduction of crowding in service (43%), communal bathrooms per 100 residents=3, private bathrooms per 100 residents=0.5, sleeping arrangements: dormitory rooms=2, ≥3 feet between beds (91%), barriers between beds=4, proportion of residents leaving/returning per day=44. COVID-19 risk factors: sleeping in shared room (>20 people), never smoked (vs. current smoker), leaving/returning to shelter everyday, lower number of bathrooms per 100 people.
Senior Homeless Population was Covid-19 Free in 3	Gombita et al. (2020), Slovakia/Hu	Unspecified - Observational cohort	All three shelters had no COVID-19 cases. COVID-19 antibody and PCR tests conducted. 1 case had history of infection. Shelters provided: semi-quarantine (quarantine during first month and limited outings for

shelter communities after adapting the Life Island model (Note)	ngary border, Slovakia/Austria border, Slovakia/Poland border		months two and three), incentives (free cigarettes and food daily), medical care (daily GP visits), free medical supplies for any diseases, media services (e.g., TV, computer; television), religious/cultural events.
Determinants of health care use among homeless individuals: evidence from the Hamburg survey of homeless individuals	Hajek et al. (2021), Germany	Prospective study	Characteristics: single (67%), widowed/divorced/married, living away from spouse (33%); presence of chronic alcohol consumption (37%); health insurance: yes (69%); mean fear of COVID-19 (range: 1 [=not at all] to 4 [severely])=1.8; health-related quality of life (QOL) (range 0 [worst] to 100 [best])=75.5; number of physician visits in previous 3 months=3.5; hospitalisation in previous 3 months: none (58%), at least one (42%). Predictors of increased number of physician visits (previous 3 months): being female, absence of chronic alcohol consumption, lower-health related QOL. Predictors of hospitalisation (in last 12 months): lower age, having health insurance, lower-health related QOL.
Prevalence and Factors Associated with Fear of COVID-19 Among Homeless Individuals During the COVID-19 Pandemic: Evidence from the Hamburg Survey of Homeless Individuals	Hajek et al. (2021), Germany	Cross-sectional	56% had no fear, 24% had a little, 9% were somewhat, and 11% were severely afraid of COVID-19. Regression analyses: younger age, absence of chronic alcohol consumption, increased perceived risk of contracting COVID-19, and higher belief that contracting COVID-19 would ruin their life was associated with fear of COVID-19. All results remained significant when accounting for missing values.
Low Barrier Tele-Buprenorphine in the Time of COVID-19: A Case Report	Harris et al. (2020), United States	Case report	Collaboration between street outreach and a low-barrier addiction clinic developed tele-buprenorphine initiations for two cases. Both cases: presented to harm-reduction specialist from streets/couch surfing; were linked to bridge clinic addiction medicine specialist for telemedicine; have history of overdoses and severe OUD; used heroin/fentanyl in the previous days; electronically prescribed naloxone/buprenorphine; educated on COVID-19 prevention.<br>Case 1: history of heroin, fentanyl, non-prescribed benzodiazepines, cocaine, methamphetamine use, benzodiazepine use disorder (BUD) and stimulant use disorder (UD), incarceration; educated on safe injection practise and HIV; ready to cease benzodiazepine use-start medically managed benzodiazepine withdrawal at fourth follow-up.<br>Case 2: history of cocaine use disorder (CUD) and mood disorder; no history of naloxone/buprenorphine treatment. Advised to get tested for HIV.

National COVID-19 Homeless Service User Experience Survey	Health Service Executive, Ireland	Report	Additional accommodation provided for social distancing, cocooning, shielding, isolation. Some accommodated in hotels; meals provided. Some PEH recommended improved quality and frequency of meals.<br>55% self-reported health good-to-excellent, 28% fair, 17% poor/very poor. 46% self-reported general health better than 1 year prior, 34% worse, 30% the same. 54% self-reported QOL as good-to-excellent, 28% fair, 18% poor/very poor. 70% felt safe/very safe, 18% somewhat safe, 11% not very safe/not at all safe. 46% felt safer than 1 year prior, 41% same sense of safety, 13% felt less safe. 54% self-reported mental health status as good-to-excellent, 27% fair, 19% poor/very poor. 29% self-reported mental health better than 1 year prior, 39% worse, 22% the same. 21% reported self-harm, suicide attempt, or suicidal thoughts in last month. 17% had suicidal ideation in last month–significantly higher in county Dublin than Galway, Limerick, Clare, and Tipperary (21% vs. 13%, respectfully). 24% of those in Dublin shielding/isolation facilities had suicidal thoughts. 55% using drugs and/or alcohol. 47% said it impacted daily activities in last year, and 35% in past month. 48% self-reported changes to overall health and well-being (including mental health) since cocooning or shielding: 45% no change, 7% didn't know. Those reporting change: 37% positive and 56% negative changes.<br>Positive changes: increased sense of safety, improved living situation, reduced drug use, improved relationships, exercise. Negative changes: poorer mental health, anxiety, depression, uncertainties regarding future housing, reduced access/ability to drug support services/exercise/cook meals, reduced family visits. Number of visits with health professionals remained same (April–June, 2020) compared to before (September–March, 2020). Engagement with key workers/case managers increased. Access to secondary health services (e.g., hospital impatient/outpatient, emergency department) lower during outbreak, except in Galway-17% increase in number of emergency department (ED) visits. Fewer visits to local health centres in Limerick, Tipperary, Clare. 16% accessing new health supports since outbreak; majority newly accessing drug/alcohol services, mental health services (online and phone support); other new supports (GP, primary care, on-site nursing, housing support, project worker support). 53% had up to date care plan, 32% did not, 15% did not know. 70% of respondents in Galway, Limerick, Tipperary, Clare had up to date care plan, compared to 35% in Dublin. 70% of respondents felt satisfied/very satisfied with services.
Surveying Tenants of Permanent Supportive Housing in Skid Row about COVID-19	Henwood et al. (2020), United States	Cross-sectional	Mental health diagnosis (59%), unaware of COVID-19 outbreak (1%), views COVID-19 health risk seriously (65%), handwashing all the time (75%), social distancing all the time (70%), pre-existing condition COVID-19 risk group (39%), flu-like symptoms in previous 30 days (4%), able to shelter in place for 14 days (55%). Proportion of PEH accessing: hygiene items (74%), food delivered (91%), medication delivered (38%), someone to check in on them (29%), something to do (16%), none of these (2%).

			Higher proportion of those housed in studio's (vs. single rooms): with a mental health diagnosis or at higher COVID-19 risk due to pre-existing condition or flu-like symptoms in previous 30 days; social distancing all the time; have access to: food deliveries, hygiene items, medication deliveries. Predictors of perceiving COVID-19 to be a very serious health risk: female gender, older age, pre-existing health conditions. Predictors of handwashing: mental health diagnosis, perceiving COVID-19 to be very serious health risk. Predictors of social distancing: staying in single-room, perceiving COVID-19 to be very serious health risk.
Pragmatic randomized trial of a pre-visit intervention to improve the quality of telemedicine visits for vulnerable patients living with HIV	Hickey et al. (2020), United States	Pragmatic randomized controlled trial	Telemedicine arranged before HIV clinic appointment (rather than face-to-face visits-unless necessary). Phone-based medical interpreters used (for primary languages not English/Spanish). Telemedicine call purpose: remind patients of upcoming appointment, give information on how to contact clinic/access in-person care, offered student enrolment, assistance identifying location to conduct phone call, identifying agenda items, care co-ordination needs from other interdisciplinary team members, medication instructions, communicate record methods, screen unmet social needs.<br>PEH: not contacted (17%); contacted (9%)-enrolled (8%), not enrolled (1%). Intervention purpose: address knowledge, structural and communication barriers to phone visits. PEH enrolled in: intervention group (7%), control group (15%). No significant differences between PEH and not experiencing homelessness attending pre-visit call.
Viral suppression during COVID-19 among people with HIV experiencing homelessness in a low-barrier clinic-based program	Hickey et al. (2021), United States	Non-randomized pre/post design, (interrupted time series)	POP-UP-low-barrier model of care developed to facilitate care for PEH with HIV during pandemic.<br>98% had SUD. 89% used methamphetamines. Mean/percentages pre-COVID-19 (10/17/2019 to 3/16/2020): visits per month to HIV primary care programme (1.6), proportion of patients visiting each month (64%), viral suppression (48%). Mean/percentages post-COVID-19 (3/17/2020 to 8/16/2020): visits per month to HIV primary care programme (1.7), proportion of patients visiting each month (58%), viral suppression (47%). 15% temporarily housed in shelter-in-place hotels; facilitated phone and in-person outreach by POP-UP navigator.
Race/Ethnicity, Underlying Medical Conditions, Homelessness, and Hospitalization Status of Adult Patients with COVID-19 at an Urban Safety-Net Medical Center-	Hsu et al. (2020), United States	Unspecified – Cross-sectional (author communication)	PEH prevalence (16%), outpatient management (12%). PEH hospitalised prevalence (1 March-18 May, 2020): non-ICU inpatients (24%), admitted to ICU w/o mechanical ventilation (16%), admitted to ICU with mechanical ventilation (16%), died (15%).

Boston, Massachusetts, 2020			
Implications of COVID-19 vaccine uptake among young adults experiencing homelessness: a brief report	Hsu et al. (2021), United States	Cross-sectional	>65% somewhat-to-strongly agreed COVID-19 vaccines would be necessary to protect health/prevent spread of COVID-19. >50% agreed that vaccines would be safe, >56% were slightly-to-extremely likely to get vaccinated when available and if recommended by a doctor. <80% believed access to PPE and primary prevention services contributed moderately-to-a great deal to vaccine uptake. 70% believed access to free COVID-19 treatment, text-based COVID-19 prevention information, ability to get vaccinated in non-regular medical settings were critical to vaccine uptake.
Impact of the COVID-19 pandemic on the homeless: results from a retrospective closed cohort in France (March-May 2020)	Husain et al. (2021), France	Retrospective serological study	Three homeless shelters-two HCAs (healthcare accommodation), one women's shelter), provide medical/accommodation PEH needs. HCA provided accommodation, medical services, residents shared: meals, bathrooms, showers. Women's shelter: bathrooms, showers, meals shared but no windows to ventilate rooms. PCR and serological tests offered to all.<br>Prevalence of: addictive behaviours (43%), alcohol abuse (28%), active chronic smoking (35%), substance abuse (6%), chronic obstructive pulmonary disease (10%), heart condition (11%), chronic kidney disease (7%), body mass index ≥30 (12%), type 2 diabetes mellitus (18%), cancer (5%), hypertension (20%), cerebrovascular disease (2%), liver disease (4%), pregnancy (2%), psychiatric disorder (17%), active tuberculosis (7%), HIV (4%), autoimmune disease (4%), ≥2 risk factors for severe SARS-CoV-2 (38%), isolation (71%), positive PCR (30%), positive serology (70%), infection attach rates (IAR; 72%). Of positive IAR cases: hospitalised (24%), infection fatalities (6%). Of COVID-19 infected individuals, 60% symptomatic. Of hospitalised cases, 71% needed oxygen therapy, 12% transported to ICU. 100% of deaths from HCA. IAR more frequent in women's shelter (91%) vs. HCA (63%); fewer risk factors for severe COVID-19 infection in women's shelter (13%) vs. HCA (50%). Median days from suggestive symptoms/positive PCR to serology testing=124 days. Hospitalised (vs. non-hospitalised) PEH from HCA more likely to be over 65 years (15% vs. 65%) or have: heart condition (4% vs. 47%), chronic kidney disease (4% vs. 24%), or 2 or more risk factors for severe COVID-19 (31% vs. 82%).
Acceptance of COVID-19 vaccine among persons experiencing homelessness in the City of Rome, Italy	Iacoella et al. (2021), Italy	Cross-sectional	Contracted COVID-19: no (85%), yes (4%), unsure (12%). 64% tested at least once (willingly). 32% did not want to be vaccinated, 4% unsure. Men more likely to take vaccine (74% vs. 59%); 30–39-year-olds least willing (46%); 60-69-year-olds most willing (85%); 53% of 40–49-year-olds and 62% of 50-59 year-olds willing to be vaccinated.
Coronavirus Disease 2019 (COVID-19) Outbreak in a San	Imbert et al. (2021),	Cross-sectional	Daily temperature checks (27 march, 2020) and symptom checks the following week. Tests performed with RT-PCR assay (6/7 April, 2020). Positive cases were not admitted to centre. Shelter closed 11 April, 2020.

Francisco Homeless Shelter	United States		26 beds within 6 feet of initial cases; 69% were occupied & deemed close contacts. Of occupied beds or close contacts, 22% returned positive. Characteristics: comorbidities (27%): most common-hypertension, congestive heart failure, diabetes. Residents tested (59%): 67% were positive, 67% were symptomatic, 52% were asymptomatic, 0.4% tested positive post-mortem. Discharge: 75% moved to I&Q hotel rooms, 99% of positive cases: moved to I&Q hotel room. Of positive cases some: had treat and release emergency visits (12%), were hospitalised (8%), died (0.4%). Comorbidity: and hospitalised (38%); not hospitalised (27%). 52% of positive cases completed case interview: the rest were not contactable.
Impacts of the COVID-19 Pandemic on Preexisting Racial and Ethnic Disparities, and Results of an Integrated Safety Net Response in Arlington County, Virginia	Irwin et al. (2021), United States	Case study	Number of unsheltered PEH increased by 91% (February-May 2020)-increasing risk of contracting virus. Two hotels rented to isolate low-income individuals unable to isolate/quarantine effectively. Fatal and non-fatal overdoses increased by 120% and 44% respectively in residents from 2019-to-2020. PEH given phones with prepaid minutes to connect/engage with telehealth.
Widespread severe acute respiratory coronavirus virus 2 (SARS-CoV-2) laboratory surveillance program to minimize asymptomatic transmission in high-risk inpatient and congregate living settings	Jatt et al. (2020), United States	Cross-sectional	Developed temporary shelter units for up to 218 PEH. PCR test used for COVID-19 testing. All tests returned negative for PEH in shelter. Two positive cases housed in designated isolation area before entering shelter.
Homeless Shelter Characteristics and Prevalence of SARS-CoV-2	Karb et al. (2020), United States	Cross-sectional	PEH (99%) tested for COVID-19 from 5 shelters: 12% positive, any comorbidity (38%), any symptoms (15%). Of positive cases: symptoms (20%), fever/hypoxia (0%). Shelters: wear facemasks (100%), daily temperature checks (100%), at least daily symptom screenings (100%: 40%-two per day), on-site meals (100%), sleeping space 6 feet apart (60%), open 24-hours (100%), communicate daily education/updates

			(20%), allow new residents (40%). Two shelters with positive cases. Positive cases less likely to have comorbidity; negative cases more likely to spend at least two weeks at shelter.<br>Shelter with highest number of positive cases: had highest census-tract population density, least proportion of residents staying for at least two weeks (58%), unable to distance six feet between individual sleeping spaces.
Successful public health measures preventing coronavirus disease 2019 (COVID-19) at a Michigan homeless shelter	Kelly et al. (2020), United States	Disease Prevention Protocol	Shelter provided accommodation, bi-daily meals. Symptom screening upon entry, social distancing, and housing in alternative areas (church/hotel). SARS-CoV-2 testing offered to all, social distancing and sheltering in place encouraged. Facemasks mandatory. Risk-based Triage: Negative test-could sleep at shelter as per usual; ≥1 symptom-sleep 6 feet apart (wearing facemasks and taking clinical assessment); ill cases-transported to emergency room. Those awaiting tests quarantined in private rooms. Two positive cases identified (both triaged ill).
Factors associated with SARS-CoV-2 positivity in 20 homeless shelters in Toronto, Canada, from April to July 2020: a repeated cross-sectional study	Kiran et al. (2020), Canada	Retrospective chart audit	Mobile outreach COVID-19 testing was available to participants, by a physician, nurse practitioner or registered nurse. Out of 17 shelters, testing acceptance range: 15-86%. Previous medical history: any chronic condition (82%), cardiovascular disease (6%), chronic lung disease (6%), HIV (3%), diabetes (9%), current smoker (55%), mental health diagnosis (27%), substance use (31%). Symptoms reported: any (10%), cough (6%), shortness of breath (<2%), fever (4%), other (4%). Of 4 shelters with more than one positive case, positive residents were significantly less likely to have health insurance card/visited another shelter in previous fourteen days.
Caring for COVID's Most Vulnerable Victims: A Safety-Net Hospital Responds	Komaromy et al. (2021), United States	Retrospective	CRU created as safe/supportive place to isolate for substance use/mental health disorders. Harm reduction, addiction treatment programmes, counselling/social work services, naloxone/methadone treatment, telehealth, buprenorphine, HIV testing, PPE supplies, cigarettes, infection controls practises, TV, clothes, meals available. Inpatient beds preserved for: CRU patients not in need of acute care but high-risk for spread of virus/medically ready for discharge after hospitalisation.<br>Characteristics: psychiatric diagnosis (79%), two or more psychiatric diagnoses (38%), active substance use-(alcohol, cocaine, opioids, methamphetamines, benzodiazepines; 42%), actively use more than one substance (18%), at least one psychiatric and substance use disorder (30%). No deaths; non-fatal overdose (3%). Discharge: substance use/mental health disorder programme (11%), family members (12%), rest to shelters.<br>Transferred to main medical centre (medical/psychiatric issues): COVID-19 symptoms exacerbating (5%), acute respiratory failure/low oxygen saturation (2%), acute cardiac issues (2%), coagulation issue (0.4%), renal issue (0.4%), medical evaluation (4%), psychiatric evaluation (3%).

Cohortation and testing of elderly homeless within COVID pademics in an urban environment - Example of a life island mission model	Krcmery et al. (2020), Slovakia	Unspecified - Observational cohort	Special shelter for elderly PEH developed to isolate residents during pandemic: Eight weeks semi-lockdown (had access to local garden). 16% did not comply with regimen-transferred to low threshold centre. All tested and negative for COVID-19 (used German Covid Ab IgM and IgG rapid test). Free cigarettes, daily meals (three per day), TV, social counselling, laundry, spiritual and educational talks were provided to encourage staying indoors.
COVID-19 vaccine access and attitudes among people experiencing homelessness from pilot mobile phone survey in Los Angeles, CA	Kuhn et al. (2021), United States	Pilot study	Housing status (night before participation): unsheltered (44%), sheltered (31%), shared/hotel (18%), other (7%). Severe/moderate psychological distress (52%) 33% saw COVID-19 as high threat. 42% had high COVID-19 protective behaviour (e.g. hand washing, following guidelines). 62% received COVID-19 information from official sources, 56% from media, 42% from personal sources. 48% reported vaccine hesitancy. Reasons for vaccine hesitancy: fear of side effects (37%), wanted more information (30%), rejected all vaccines (27%), want more people to take it first (20%), part of risk group & want more information (17%), don't believe vaccine will protect them (17%), other (17%). 19% offered vaccine, 11% accepted. Of those not offered vaccine: 51% would take, 32% would not, 17% did not answer. No difference in vaccine hesitancy between demographics.<br>Hesitancy associated with: lower psychological distress scores, lower perception of COVID-19 as a threat, were not more likely to engage in COVID-19 prevention behaviours, but were more likely to avoid touching their face, less likely to trust COVID-19 information from official sources, and mass media, not more likely to trust information from friends, family, or social media. Less likely to be vaccine hesitant if they had high perception of COVID-19 as threat or trusted official sources. More likely to be vaccine hesitant if: they performed highly protective COVID-19 behaviour, trusted personal contacts (remained significant when compared to official and mass media sources separately).
Elevated Mortality Among People Experiencing Homelessness With COVID-19	Leifheit et al. (2021), United States	Cross-sectional	9% of included jurisdictions (n=76) had data on PEH. Case fatality rate (CFR) range: 0.3-4.8% (vs. 0.6-2.5%: general CFR). Overall CFR: for PEH (2.1%), for general population (1.6%). In Los Angeles, CFR were higher among PEH under 65 years old (vs. general population). The opposite true for those over 65.
The pandemic and homeless people in the Turin area: The level of housing adequacy shapes	Leonardi et al. (2021), Italy	Case study	First few weeks of pandemic: shelters open at night. PEH occupied public spaces during the day, returned to shelters, risking spreading of virus. Loss of places for PEH to volunteer/work. Soup kitchen remained open, delivering food. Shelter had rotational system, collective living, crowding – facilitated virus transition. Changes implemented: stopped rotation system and new admissions (from 12 March 2020). Shifted to 24 hour operation. Negatives: more sleeping rough, with no supports. Confusion and lack of

experiences and well-being			I&Q facilities put residents and staff at risk. After first cases, frontline worker support shifted to outdoor support, providing food, medication and information. Surveillance of shelter from outside, to prevent PEH leaving. Some symptomatic residents transferred to COVID-19 specific hospital. Shelter disinfected after one week. Shift to 24/hr services, abolished rotation, space arrangements fostered sense of community, stability, increased privacy in shelters. Residents sought cleaning supplies for living spaces, felt improved sense of safety, independence, wellbeing. Those with Housing First projects had more agency, power and control over own health/lives, provided phones.
COVID-19 vaccine hesitancy among persons living in homeless shelters in France	Longchamps et al. (2021), France	Cross-sectional	Overall characteristics: living alone (82%), living with a partner (19%), living with children (35%), length in shelter: ≤1 month (11%); 1 month-1 year (49%); >1 year in shelter, social support outside shelter (95%), depression (28%), chronic disease (26%); fear of SARS-CoV-2 infection (67%), trusted official information on COVID-19 (76%). Health literacy: low (49%); intermediate/high (51%). Source of COVID-19 related information: TV (63%); social media (84%). Unwilling to be vaccinated (41%): of these, 71% did not want vaccine, 29% did not know.<br>Factors associated with vaccine hesitancy: being female, living with a partner (vs. alone), living with children, region of birth, French/legal residence, low health literacy. Factors associated with vaccine hesitancy in multivariate logistic regression analysis: being female, living with a partner, French/legal residence, low health literacy.
Seroprevalence of SARS-CoV-2 antibodies among homeless people living rough, in shelters and squats: A large population-based study in France	Loubiere et al. (2021), France	Cross-sectional seroprevalence	Seroprevalence, IgM, IgG testing offered to all. 91% eligible.<br>Achieved lower secondary education or higher (48%), live with someone (45%), had health insurance (70%), had no financial resources (43%), did not have a job (83%), experiencing homelessness for twelve months or less (27%), in a shared room/area (56%), in a private room/area (44%), moved accommodation since COVID-19 (37%); consumed tobacco (53%), alcohol (23%), illegal drugs (17%); had at least one comorbidity (53%), psychiatric and substance use disorder comorbidities (24%). COVID-19 risk factors: obesity (7%), diabetes (8%), cancer (2%), chronic respiratory pathology (9%), cardiovascular pathology (14%), chronic renal failure (2%). IgG positive=5%, IgM positive=2%, positive for both=2%. Seroprevalence=6% and significantly highest among European Typology of Homelessness and housing exclusion 2 (ETHOS 2; in emergency shelters/slums). 57% of COVID-19 positive PEH spent more than one month in emergency shelters (vs. 30% with negative tests). Seroprevalence lower in tobacco consumers, those with psychiatric/substance use disorder comorbidities symptomatic PEH.
Screening of SARS-CoV-2 among homeless people, asylum-seekers and other people living in	Ly et al. (2021), France	Cross-sectional	Three shelters (A,B,C) had special units for high-risk sedentary PEH with high levels of poverty, poor hygiene, AUD, mental health condition, chronic diseases. All required to remain under strict lockdown, within shelter (from 17 March, 2020). Prevention strategies: avoiding gatherings of people, wear facemasks, social distancing, washing hands (minimum 20 seconds), practising cough etiquette, avoiding touching eyes/nose/mouth with unwashed hands. All tested and isolated for COVID-19 until tests results

precarious conditions in Marseille, France, March-April 2020			returned. Some residents moved among homeless shelters to hotels to avoid overcrowding. Staff available: management, social workers, nurses, cleaning, catering and security. Positive cases: moved to special COVID-19 isolation for PEH or went to single isolation room with strict isolation measures.<br>Homeless shelters with access to: bathrooms/toilets-shared (63%), private (38%); shared kitchen (100%); open space-large terrace (88%), cultural hall (25%), none (13%); medical care and behavioural health resources available-at shelter (38%), on demand (63%); 63% of accommodations did not have emergency or long-term beds; total capacity range: 10-310; number of emergency beds range: 50-280; number of long-term beds range: 14-50. Acceptance for COVID-19 testing among PEH (75%) significantly lower than employees of shelter (89%). Proportion of PEH: in study (59%), tested (76%), positive (9%). Presence: at least one symptom (24%), cough (13%), rhinorrhea (12%), dyspnoea (7%), sore throat (6%), fever (2%), death (0%).
Variations in respiratory pathogen carriage among a homeless population in a shelter for men in Marseille, France, March-July 2020: Cross-sectional 1-day surveys	Ly et al. (2021), France	Repeated cross-sectional	Special units available for high-risk sedentary PEH with high levels of poverty, poor hygiene, alcohol use disorder, mental health condition(s) and chronic diseases. Virus spread prevention strategies: strict lockdown, avoiding gatherings, wearing facemask, social distancing, washing hands, cough etiquette, avoiding touching eyes/nose/mouth. Positive SARS-CoV-2 cases moved to isolation facilities for PEH or kept in single rooms at shelter with strict isolation measures. All residents under strict lockdown (March 17-May 11, 2020).<br>All participants completed nasal sampling at least once (about 56% of PEH). 13% tested 3 times. 8% of samples positive for SARS-CoV-2. March-early April symptom prevalence: at least one respiratory symptom (32%), cough (15%), rhinorrhoea (12%), dyspnoea (9%), sore throat (5%), fever (8%). Late April symptom prevalence: at least one respiratory symptom (12%), cough (5%), rhinorrhoea (5%), dyspnoea (1%), sore throat (2%), fever (1%). Mid-July symptom prevalence: at least one respiratory symptom (6%), cough (3%), rhinorrhoea (0%), dyspnoea (0%), sore throat (0%), fever (1%).
Response to the COVID-19 pandemic among people experiencing homelessness in congregant living settings in San Diego, CA	Marquez et al. (2020), United States	Unspecified – no methods reported	Hotel rooms and free meal plans. Sheltered and unsheltered (by outreach team) PEH relocated to convention centre. At least daily screening (temperature checks, verbal questionnaire's), washing/hand sanitising upon re/entry to shelter, wore facemasks, clean/sanitise surfaces, social distancing, evaluating/isolating symptomatic cases, provided bi-hourly (8a.m.-5p.m.) public safety announcements. Collaboration between local healthcare and PEH service providers enabled pre-emptive COVID-19 testing. Positive cases moved to isolation areas within medical unit. 10 congregate living shelters tested residents (16 April, 2020-5 August, 2020). 0.9% tested positive.
COVID pandemic as an opportunity for improving mental	Martin et al. (2021), Spain	Longitudinal	PEH not allowed to sleep on streets of Salamanca during initial lockdown period. Pandemic forced implementation of assistance plan and primary care service for PEH in Salamanca, providing: access to psychologists, psychiatrists, social educators, sociosanitary, access to psychopharmaceutical

health treatments of the homeless people			interventions/medical assistance, shared rooms for 2-10 persons, meals and shared indoor/outdoor spaces, residents not allowed to leave shelter, residents complied with government rules/protocols, study completed with full PPE and use of COVID-19 prevention strategies, 50 psychiatric visits made between 7 occasions research team was on-site (27 March-5 May 2020).<br>All participants: mental health disorder (63%), symptoms of mental health disorder w/o diagnosis (19%), no differences in demographic information (those with mental health diagnosis vs. w/o), SUD (33%: rest had another psychiatric diagnosis), dual diagnosis of SUD and some other psychiatric diagnosis (22%), contacted psychiatric services previously (11%), prescribed psychopharmaceutical (almost 53%). Participants diagnosed with psychiatric condition: substance abuse history (53%); family substance abuse history (29%); current substance abuse (53%): alcohol (24%), cannabis (18%), cocaine (6%), opiates/methadone (6%); history of psychiatric hospitalisation (29%); no gender differences among variables. Impact of centre-visited: once (56%), twice (15%), three times (19%), four times (11%). Mental health diagnosis increased (63%): of these, 54% SUD, 47% another psychiatric condition, 29% an anxiety disorder, 12% affective disorder, 18% psychotic disorder. Psychopharmaceutical prescriptions increased from 59% to 82%. Psychopharmaceutical prescriptions before vs after presenting to centre: antipsychotics (12% vs. 29%), benzodiazepines (24% vs 24%), antidepressants (24% vs 29%). Emergency visits significantly reduced before vs. after presenting to centre (24% vs. 6%). None diagnosed with COVID-19.<br>Discharge: individualised plans supported for re-integration to community (100%), to a mental health service (37%): of these, 40% to outpatient mental health, 50% to outpatient drug clinics, 10% to therapeutic community for drug cessation/rehabilitation for SUD. Those with mental health disorder referrals: day centre for long-term stay and linkage with mental health resources (29%), accessed release salary and bought a home (6%), shelter for people living with HIV/acquired immunodeficiency syndrome (AIDS) or assisted with application for house rental (65%). Centre closure: 4% left outpatient drug-clinic and refused follow-up or relapsed on cocaine or requested discharge from therapeutic community after 10 days.
COVID-19: A catalyst for change in telehealth service delivery for opioid use disorder management	Mehtani et al. (2021), United States	Quality Improvement Program	PEH transferred to I&Q sites if SARS-CoV-2 positive. Addiction Telehealth Program (ATP) developed to address SUD needs in I&Q hotels. Nurses on-site, alcohol/cigarette use permitted in hotel rooms (if delivered). Needles, syringes, related equipment available on request for harm reduction. Clients contacted via telehealth, provided a plan, delivered same day medication. Some started buprenorphine treatment for unmanaged OUD, provided with verbal instructions to carry out at home, follow-up calls made. Motivational interviewing was only psychosocial treatment. Uninsured persons helped with accessing medication. Overall, no overdose deaths recorded: one case of non-fatal opioid overdose.<br>Reason for ATP consultation: alcohol use (42%), opioid use (32%), stimulant use (17%), cannabis use (7%), gamma-hydroxybutyrate use (2%). Among I&Q telehealth buprenorphine prescribed guests: 58% never previously prescribed treatment. Homeless status: sheltered (75%), unsheltered (17%), incarcerated (8%).

			COVID-19 status: positive (17%), close contact (25%), under investigation/symptomatic (58%). Opioid use disorder treatment history: buprenorphine (17%), methadone (33%), none (58%). Currently using: heroin (67%), fentanyl (42%), opioid pain pills (0%). Route of opioid use: injection (42%), intranasal (25%), inhalation (33%). Discharge: 33% self-discharged with no reasoning.
Establishment of Isolation and Non congregate Hotels During COVID-19 and Symptom Evolution Among People Experiencing Homelessness-Atlanta, Georgia, 2020	Montgomery et al. (2021), United States	Cross-sectional	PEH contracting COVID-19 relocated to either newly established isolation or non-congregate hotel. <u>*Isolation hotel:*</u> access to residential assistants (RAs). RAs supervised isolation/PEH outdoor breaks. RAs phoned PEH bi-daily: assessing personal needs, well-being, self-reported temperature/symptoms. Medical staff on-site 20 hours per day: conducting in-person temperature/symptom checks once daily; evaluate/triage medical needs. Emergencies addressed via emergency medical services; non-emergency clinical, mental health, prescription needs addressed via telehealth. 43% referred from community, 57% from hospitals. Median time from testing to date of admission=3 days, median length of stay=12 days. Community referrals: 39% symptomatic, 32% pre-symptomatic. Hospital referrals: 93% symptomatic, 7% pre-symptomatic. 83% reported symptoms at least once. Ever symptomatic community referrals=66%. Self-reported COVID-19 symptoms: cough (77%), headache (63%), muscle aches (62%), shortness of breath (50%), fever (45%), diarrhoea (45%), sore throat (37%), abdominal pain (35%), vomiting (14%). 89% had at least one symptom, 23% had all 3 symptoms, 11% had no typical symptoms (subjective fever, cough, shortness of breath). 57% had at least one gastrointestinal (GI), 8% had all 3, 43% had no GI symptoms (abdominal pain, diarrhoea, vomiting). 52% had at least one typical and one GI symptom. 30 emergencies recorded. 48% referred to emergency shelters, 15% linked with inpatient substance use treatment services, 4% self-discharged. 3% transported and admitted to hospital/other residential non-psychiatric medical facility, 3% discharged to residential project/halfway house with no homeless criteria, 2% refused housing placement and returned unsheltered. <u>*Non-congregate hotel:*</u> All expected to follow social distancing, wear facemasks, hand hygiene guidelines. Medical staff addressed: telehealth, in-person clinic visits, prescription refills, other medical/behavioural needs. Positive clients: transferred to isolation hotel/medical facility. Clients admitted in hotel until: attaining permanent housing plan; significant behavioural issue; self-discharge. Clients admitted for median of 18 weeks. 61% discharged to permanent housing/remained admitted to permanent housing plan; 25% to location not purposed for habitation; 11% to temporary housing; 3% linked with medical care site; 1% to prison/juvenile centre.
Hand hygiene during the COVID-19 pandemic among people experiencing homelessness-	Montgomery et al. (2021), United States	Qualitative	44% experiencing homelessness for the first time. PEH practised hand hygiene, accessed water in shelter/public area bathrooms. Challenges: regular access to water/water source (due to location and public toilets access), hand sanitizer, soap, wet wipes; some spit on hands (due to lack of access to hand hygiene). Sheltered/employed PEH more likely to access regular water sources. Unsheltered PEH more likely to rely

Atlanta, Georgia, 2020			on hand sanitizer, bottled water, disinfectant wipes for hand hygiene. Drying hands: some air dried/wiped their hands on their clothes; some set time slots for washing hands-determined by availability.<br>*Barriers to hand hygiene:* lack of facilities (96%); lack of supplies (74%); mental health/substance use, financial, instability, needs prioritising (safety), or if job made it difficult to get appropriate breaks to use handwash facilities.<br>*Factors increasing barriers to hand hygiene:* city/state closures (lockdown), increased demand/prices for supplies.<br>*Factors lessening barriers to hand hygiene:* increased number of non-profit organisations/people distributing hygiene supplies to sheltered/unsheltered locations. Some accessed shampoo, lotion, razor, toothbrush, combs. Having place to store items was challenging (e.g. bag)-items stolen when sleeping.<br>*Financial barriers to hand hygiene:* restaurant attitudes, no funds to purchase goods from shop with toilet, no access to toilet.<br>*Instability barriers to hand hygiene:* unsheltered, access to water, having a bed, living outside, constantly moving. Some neglected self-care/hygiene practices. PEH more concerned with shelter safety-not getting shot/physical harm (than practising hand hygiene).<br>*Supporting hand hygiene:* connection to supplies/facilities (84%), affordable housing/employment, location to practise handwashing, hand hygiene education.<br>80% saw hand hygiene information; 58% knew SARS-CoV-2 prevention strategies. PEH open to hand hygiene education: others felt it was common knowledge/unnecessary. Most interested in understanding how/where to access supplies. One participant felt there should be sheltered/unsheltered specific information distributed. Preferred method for communicating hand hygiene information: advertising messages on public transport/around shelters, electronic messaging, portable formats. Health communication issues: six feet social distancing for certain environments, crowding was barrier to practising hand hygiene/social distancing/general COVID-19 prevention guidelines.
Correlates of COVID-19 Vaccine Hesitancy among a Community Sample of African Americans Living in the Southern United States	Moore et al. (2020), United States	Cross-sectional	Those who were vaccine resistant (33%) were more likely to have experienced housing insecurity due to COVID-19 compared to acceptant (7%) and hesitant (10%) participants. Housing insecurity accounted for 8% of variation in vaccine resistance and was associated with 7-fold increased odds of vaccine resistance.
Assessment of SARS-CoV-2 Infection Prevalence in	Mosites et al. (2021),	Unspecified – Cross-sectional	Testing offered to all. Testing used: RT-PCR. Positive cases transferred to hospitals/predesignated community isolation areas. 79% tested experiencing homelessness: 25% positive.

Homeless Shelters Four U.S. Cities, March 27-April 15, 2020	United States	(author communication)	
Pandemic Planning in Homeless Shelters: A pilot study of a COVID-19 testing and support program to mitigate the risk of COVID-19 outbreaks in congregate settings	O'Shea et al. (2021), Canada	Pilot study	Collaboration between local public health unit, government, shelters, shelter health network (March, 2020): increased number of beds by 16% (across shelters, three hotels), enabled physical distancing between shelter beds, reduced risk of crowding, recreational centre re-purposed to temporary isolation centre. Medical programme used: rapid COVID-19 testing, spaces within shelters for short-term isolation, two-week isolation of positive cases. Those awaiting COVID-19 results, isolated in single rooms in-shelter. Positive cases (not needing hospitalisation) moved to isolation centre. Transportation with infection controls measures (e.g. barrier between driver and client, facemasks, face shield, gloves) used for transfers to isolation centre. Shelter residents advised not to travel between shelters. Mask wearing enforced in common areas of shelters (April 18, 2020).<br>17% diagnosed with rhinovirus/enterovirus (before 17 April, 2020). 2% positive for COVID-19 (after 17 April, 2020). 1% positive for COVID-19. No secondary COVID-19 spread linked to positive case from follow-up contact tracing/testing.
"They already operated like it was a crisis, because it always has been a crisis": a qualitative exploration of the response of one homeless service in Scotland to the COVID-19 pandemic	Parkes et al. (2021), UK	Qualitative exploratory study	Drop-in centre created (January 2020) for those at risk of or experiencing homelessness. Available: café, shower facilities, groups/social activities, support groups (e.g. women's/fitness), psychosocial programme.<br>*Initial changes:* unsure of opening/closing hours of service, where to go, how to get appointments, closure of café (caused reduction in available supports), service contacted PEH. Some felt isolated, confused, need supports with depression/anxiety. Centre remained open for one-to-one support. Other supports initially reduced, client population increased, level of support provided increased.<br>*Disrupting routines:* closures disrupted daily lives/routines. Centre remained open for one-to-one appointments but clients lost the social, comfort and safety elements of centre.<br>*Staying in touch:* staff ensured clients had access to accommodation, food, prescriptions, phones, and were able to shield safely. Emotional/practical support via phones/online. Phones with data distributed: enabled communication, feelings of dignity/care/calmness for clients, supported mental health.<br>*Socially distanced in-person support:* services more stable (May 2020)-practical and psychological telephone/online support available, continued support for those availing of services before pandemic. Limited numbers allowed in centre, some missed appointments or turned up w/o appointment, difficulties maintaining social distancing.<br>*Balancing risks and benefits:* staff limited numbers, enhanced cleaning, enforced strict rules, encouraged wearing facemasks. Hot meal takeaways, food parcels provided (from May, 2020). PEH socialised after meals despite physical social distancing. Emotional support/crisis intervention provided alongside

			distributing food parcels. Hotels/other city organisation residents accessed food. Takeaway meal provision stopped-foodbank services/food deliveries commenced.<br>*Challenges of distanced communication:* support via phone positively impacted client wellbeing: some struggled with reaching out/found it robotic.<br>*Harm-reduction developments:* location and non-medicalised environment of centre facilitated: engagement, improved/internal injecting equipment provision (IEP) service, access to naloxone/health/substance use treatment, mobile van services (IEP) ceased fore pandemic, administrative/legal barriers to naloxone provision removed, enhanced opioid substitution treatment (OST) provision; same-day prescriptions/titration available within health clinic (since 1 April, 2020), opioid treatment readily available-limited alcohol harm-reduction services.<br>*Differences:* huge benefits from support groups. Clients developed connections, trusting relationships with staff. Some PEH regained care of children, improved physical health, well-being. Centre was instrumental in these changes.<br>*Change:* long-term changes-online groups, additional outreach support for those shielding, looked for client feedback.
"You know, we can change the services to suit the circumstances of what is happening in the world": a rapid case study of the COVID-19 response across city centre homelessness and health services in Edinburgh, Scotland	Parkes et al. (2021), UK	Rapid case study	*Mental health:* exacerbated, reduced supports, increased loneliness/social isolation. Frustrations among PEH in COVID-19 high-risk category. Lack of supports, companionship, friendship in hotels. Depression increased for some. Some city services open, phone/online support available.<br>*Substance use: s*ome ceased antipsychotic medication, valium/illicit benzodiazepine, alcohol and cocaine use. Some used alcohol/drugs to cope with depression. Some reduced substance use, others increased alcohol consumption/cannabis use. Concerns with supply of street Valium-pushing them toward prescription medication. Emotional challenges from lockdown/isolation/lack of support increased substance use. Increase in prices, availability, use of street drugs. Increased engagement with needle exchange, opioid substitution programmes.<br>*Positive housing impacts:* PEH on streets ”sofa surfers” rapidly rehoused, identified newly PEH.<br>*Negative housing impacts:* victimisation-too many with complex needs in one hotel. Ongoing housing support suspended, unable to get social care, lack of PPE meant lack of visits, increased number of PEH/young PEH (family relations breakdown).<br>*Negative access to services:* exacerbated issues accessing services: no face-to-face contact/access to GPs, nurse appointments cancelled, lack of wound/mental health/dentistry services, non-COVID-19 issues from rejected A&E services. Those with problematic alcohol consumption-struggling with lack of resources, difficulty complying to social distancing, online/phone supports available to those with equipment only.<br>*Positive access to services:* easier to access some healthcare providers/medication, easier access to prescription medication/OST (for OUD), naloxone, IEP, rapid OST prescription multi-disciplinary health

			outreach service access. Naloxone home provision and setting for opiate replacement therapy (via mobile van), phone calls with clients increased, PEH provided phones.<br>*New sites for harm reduction:* managed alcohol programmes (MAPs) developed to address lack of harm reduction services. Safer client outreach models-quicker/easier access to prescription medication. Some availed of OST for first time.
The role of homelessness community based organizations during COVID-19	Pixley et al. (2021), United States	Mixed methods	*Increase in needs:* urgent need for: PPE, housing, access to healthcare, bathrooms, running water, food, medical attention, SARS-CoV-2 screening and surveillance, withdrawal support, stress management, mental health services. Lack of place to clean/shower negatively impacted dignity, hygiene, privacy. Increase in domestic violence, depression symptoms. Shift to online telehealth services (except distribution of toiletries, diapers, food vouchers)<br>*Organisational challenges and changes:* shift to online services reduced rapport building for service users; challenges accessing PPE; slowed housing support for those leaving prison (slowed court proceedings); extended meal hours; takeaway meals; daily temperature checks/surveillance; supporting alternate accommodation for high-risk individuals; increased privacy/sense of independence in non-congregate shelters facilitated acceptance of substance use treatment services; I&Q centre for PEH.<br>*Emergency management issues:* no hotel programme sheltering vulnerable populations in the future, food availability reduced, service hours changes, travel costs increased, shelters closed, accommodation not fit for purpose, under-reported health concerns of extreme temperatures for PEH, more newly PEH presenting.
Sociodemographic characteristics and transmission risk factors in patients hospitalized for COVID-19 before and during the lockdown in France	Rahi et al. (2021), France	Observational retrospective study	Total PEH testing positive for SARS-CoV-2=2%; before lockdown=1%; during lockdown=5%. Total living in disadvantaged conditions (collective housing and PEH) testing positive for COVID-19=6%; before lockdown=4%; during lockdown=12%.
Assessment of SARS-CoV-2 infection through rapid serology testing in the homeless population in the	Ralli et al. (2020), Italy	Cross-sectional	Screened for COVID-19 symptoms, referred to local hospital for further evaluation (if symptoms present). All tested for immunoglobulin G (IgG) and immunoglobulin M (IgM), and had access to facemasks, gloves, and hand hygiene gels.<br>Negative results (98%). IgG positive (1%) and IgM positive (1%). Positive cases immediately referred to hospital for further evaluation.

City of Rome, Italy. Preliminary results			
Asymptomatic patients as a source of transmission of COVID-19 in homeless shelters	Ralli et al. (2020), Italy	Cross-sectional	Performed rRT-PCR nasopharyngeal swab tests, daily symptom screening/body temperature monitoring for all. Prevention measures: facemasks (mandatory), daily health education, hygiene protocols, social distancing. Of positive cases (4%): 75% asymptomatic, 25% had symptoms including fever, diarrhoea, cough.
Criminal Justice-Involved Women Navigate COVID-19: Notes From the Field	Ramaswamy et al. (2020), United States	Qualitative	31% marginally housed. 37% of interviewee's experiencing homelessness; some stayed with family, lived unsheltered, in cars, encampments, other unsuitable arrangements. Some living arrangements prevented participants from practising social distancing. One case not allowed to quarantine, forced to wear bandana (as mask), has chronic obstructive pulmonary disease (COPD), relies on others (e.g., nurse) to transport belongings, dry goods, fears contracting COVID-19, has inadequate access to food, asked to leave family members house for having cough/runny nose.
A medicalized hotel as a public health resource for the containment of Covid-19: more than a place for quarantining	Ramírez-Cervantes et al. (2021), Spain	Descriptive	Medicalized hotel created in Madrid to reduce SARS-CoV-2 spread: sanitation, medication, medical support provided. Social distancing followed. Access to psychological/medication supports, laundry, cleaning, individual rooms. 32% had no permanent address (experiencing homelessness or eviction). Those experiencing homelessness/requiring social support worked established discharge plan.
Outbreak of SARS-CoV-2 infection at a large refugee shelter in Toronto, April 2020: a clinical and epidemiologic descriptive analysis.	Redditt et al. (2020), Canada	Cross-sectional	Nasopharyngeal swab/PCR used: 95% tested (42% positive). Positive cases transferred to COVID-19 isolation centre. Within 14 days post-testing: 13%=asymptomatic; rest had at least one symptom. Symptoms: headache (58%), new loss of taste (42%), fever (33%), myalgias (25%), sore throat (25%), cough (21%), shortness of breath (4%), chills (13%), new loss of smell (29%), new nasal congestion (25%), diarrhoea (13%), malaise (21%), dizziness (17%), nausea/vomiting (8%), chest pain/tightness (17%), other (29%). No deaths, discharges to ICU, intubation. One case discharged to COVID-19 isolation facility.
Social Work during the COVID-19 Crisis: Responding to Urgent Social Needs	Redondo-Sama et al. (2020), Spain	Qualitative	Many social resources for PEH closed or limited their schedules: PEH w/o access to showers, food. Limited places for PEH to shelter (loss of housing). Hotels eventually housed PEH, decreased spread of virus. Some PEH newly identified.
Factors Associated With Symptoms of Depression and	Riley et al. (2021),	Cross-sectional	38% experiencing homelessness. Recently experiencing homelessness predicted higher depression and anxiety scores.

Anxiety Among Women Experiencing Homelessness and Unstable Housing During the COVID-19 Pandemic	United States		
Using a 'Big Events' framework to understand emergency department use among women experiencing homelessness or housing instability in San Francisco during the COVID-19 pandemic.	Riley et al. (2021), United States	Cross-sectional community-based study	All participants women experiencing homelessness; 44% sleeping on street or in shelters. Experiencing homelessness during the pandemic associated with higher number of ED visits.
The keys to control a COVID-19 outbreak in a haemodialysis unit.	Rincón et al. (2020), Spain	Analytical observational study	Testing following outbreak in haemodialysis clinic. All underwent PCR testing. Guidance around rapid identification, hygiene, and isolation of cases followed. Rooms for isolation provided. Among PEH/living in a nursing home: 25% positive, 8% negative. PEH/living in nursing homes was a risk factor and displayed higher prevalence of positive cases. After study screening, 50% of newly positive cases were PEH.
Homelessness during COVID-19: Challenges, Responses, and Lessons Learned from Homeless Service Providers in Tippecanoe County, Indiana	Rodriguez et al. (2021), United States	Community-based participatory research	Non-profit PEH organisation provided shelter, housing services, case management, three meals daily, access to: showers, phones, toiletries. Referred to residents as “guests”. *Individual challenges:* PEH have increased risk for COVID-19 (ongoing health issues/no place of residence). Concerns for PEH stress levels, supports available, routine, mental health, ability to relax in usual locations during the day due to pandemic. Some services reduced/stopped providing substance use treatment services/counselling-behavioural issues and relapse rose. Lack of access to showers/washrooms/hygiene stations made it more difficult to follow recommended/basic hygiene practises. Closure of businesses/non-essential organisations: created lack of safe/comfortable environments available, clothes, meals, food donations. Being in close proximity to one-another, sharing cigarettes/food, touching each other in congregate/shelter settings exacerbated difficulties following COVID-19 prevention strategies. PEH: did not see virus as threatening; uninterested in departmental communications around the virus; feared

			movements being restricted/isolated from others. Some found alternative ways to quarantine, tackle being away from family/friends. Service provision moved to telehealth. Some PEH had no access to phones. When provided free phones, further barriers: no access to charge/store electronic equipment or email/social media accounts. Staff felt organisations failed to communicate need-specific information to clients w/o technological equipment.<br>*Organisational:* did not feel prepared but responded appropriately/remained open to meet PEH needs. Some shelters closed operations, making it harder to contact/have ongoing supports for PEH. PEH w/o contact details left w/o medication management. Social distancing, following recommended COVID-19 prevention strategies and reminders took place in communal spaces and meal times: less contact between guests and staff. Information re: procedures/expectations of PEH communicated in face-to-face group shelter announcements. PEH struggled to socially distance, wear facemasks: forcing some organisations to reduce/cease service provisions (e.g. meal services). Some organisations experienced struggling to keep up with costs of pandemic (particularly food, facemasks) or enforced strict facemask policies. Others preferred to see the bigger picture of keeping PEH off the streets, instilling prevention strategies slowly. Some PEH isolated awaiting results and testing only available through emergency services. Fevers >37.8°C, tested for COVID-19, transferred to hospital by health department-isolated if positive. More PEH required access to shelters, (due to unemployment, reduced incomes): hotel rooms provided for high-risk clients/soon to be housed individuals.<br>*Community:* donated money. Housing hotline developed through collaboration between homelessness agencies. Some stopped taking new admissions. One community developed place of shelter during day (with showers, food, furniture).<br>*Policy-level:* some PEH had no location to practising hand hygiene/access to bathroom.
Seroprevalence and risk factors of exposure to COVID-19 in homeless people in Paris, France: a cross-sectional study	Roederer et al. (2021), France	Cross-sectional seroprevalence study	Food distribution sites: 42% in shelters or streets/camps. Worker residences: 34% sharing room with 2-5 other people. Emergency shelters: 59% shared room with second person, 4% share room with more than five people. Seroprevalence among PEH (overall=58%): workers residence (89%), emergency shelter (50%). Seroprevalence PEH ranges between same service type: emergency shelters (23-62%), workers residences (82-94%). Likelihood of being seropositive 12 times higher for those in workers residences, 1.7 times higher in emergency shelters compared to food distribution sites.
Characteristics of COVID-19 in Homeless Shelters: A community-Based Surveillance Study	Rogers et al. (2021), United States	Cross-sectional, community-based surveillance	Testing (RT-PCR, lab-developed test/research assays) offered to all. Handwashing and social distancing practised by all, some shelters had shortage of hygiene resources. Surge testing conducted in previously positive shelters. Kitchen, TV room, sleeping halls, isolation rooms, showering facilities, rotating staff, communal floor mats available in at least one shelter.

			PEH (75%): 70% asymptomatic, 30% symptomatic. Private/shared rooms available (36%). After shelter closures, all residents moved to other shelters to reduce overcrowding. 79% of shelters used for routine surveillance testing; 27% of shelters for surge testing only. Capacity range: 45-275. Mid-nasal samples obtained from/self-collected by residents. PEH tested (89%): routine surveillance (90%), surge testing (85%), positive (86%), negative (89%). PEH sleeping in communal area for past 7 days (78%): routine surveillance (81%), surge testing (68%), positive (85%), negative (78%). PEH sleeping in private/family room for past 7 days (22%): routine surveillance (19%), surge testing (32%), positive (14%), negative (22%).
SARS-CoV-2 screening among people living in homeless shelters in Brussels, Belgium	Roland et al. (2021), Belgium	Cross-sectional	National officials moved symptomatic PEH to emergency shelters/hotels. Shelters with large numbers of suspected cases given testing priorities. Positive results notified immediately to doctor or case. Homelessness/human rights organisations collaborated with shelter doctors, co-ordination teams to establish isolation, testing, protection measures (facemasks, hand gel), contact/source trace analysis. Homelessness organisation moved PEH from public locations to isolation centres. All residents offered PCR testing.<br>Access to care: yes (35%), no (31%), urgent medical card (29%), unknown (5%). Symptoms: no (90%), yes (10%). Symptoms reported: fever (3%), cold (4%), cough/shortness of breath (3%), sore throat/head (4%), worsening of symptoms (8%). Previous chronic respiratory condition: no (91%), yes (9%). Sharing room with COVID-19 positive case: yes (5%), no (79%), unknown (16%). PCR results: positive (5%), negative (95%). COVID-19 prevalence was higher at start of study (April 27; week 1=20%). Next highest week of prevalence=week 4 (5%). Decrease in number of positive cases after week 1 (those with symptoms: 8% worsened). 3% of positive cases had symptoms. COVID-19 risk factors: having urgent medical card, sharing room with positive case or unsure if sharing room with a positive case. Positive cases had lower prevalence of previous chronic respiratory disease.
Citywide serosurveillance of the initial SARS-CoV-2 outbreak in San Francisco	Routledge et al. (2021), United States	Pilot study	Seroprevalence among PEH: 11%.
A model of disparities: risk factors associated with COVID-19 infection	Rozenfeld et al. (2020), Spain	Cross-sectional	Higher risk of COVID-19 infection was associated with higher housing insecurity.
COVID-19 Susceptibility and	Sachdev et al. (2021),	Cross-sectional	2.7% of persons co-infected (HIV and COVID-19) stayed in shelter, 7% in single room occupancy hotel, 3% experiencing street homelessness, 28% not housed but did not specify further.

Outcomes Among People Living With HIV in San Francisco	United States		
Congregate Shelter Characteristics and Prevalence of Asymptomatic SARS-CoV-2	Samuels et al. (2020), United States	Cross-sectional, multicentre cohort study	Shelters remained open during pandemic; positive cases transferred to COVID-19 isolation hotel. Nasopharyngeal swab testing conducted. 40% of shelters had positive cases; of shelters with positive tests, 29% of residents were confirmed positive and shelters were more likely to have higher population density census tract, be at capacity and allow new residents. One shelter had regular turnover: 58% of their residents stayed over two weeks. 20% of positive cases reported symptoms.<br>In all shelters: facemasks worn, daily temperature checks, symptom screening, on-site meals, open 24 hours. 60% of shelters ensured social distancing for sleep and allowed new residents. 20% of shelters provided daily education/updates re: COVID-19. Overall: medical comorbidities (38%), symptoms (15%), positive and reported symptoms (20%), fever (0%), hypoxia (8%).
Finding stability amidst the COVID-19 pandemic: The impact of emergency temporary housing for people who use drugs	Scallan et al. (2021), Canada	Case series	Both cases had OUD and stimulant UD and were temporarily housed in hotels during COVID-19.<br>Case 1: prescribed therapeutic dose of methadone (currently using methamphetamines, cased fentayl consumption; pre-March, 2020); slept outdoors/at drop-in site after being ejected from shelter-socially isolated/limited contact with husband, recommenced fentanyl use/increased methamphetamine use; continued methadone treatment (but missed more doses); refused a place to shelter due to substance use; healthcare providers/community groups provided hotel room for her/husband; eliminated substance use; found hotel motivating, provided stability, allowed her to reconnect with children, work toward permanent housing.<br>Case 2: chronic HCV infection and HIV; unable to achieve therapeutic dose of methadone for opioid agonist therapy (OAT) previously; took HIV antiretroviral therapy; got room in hotel (during COVID-19); methadone delivered daily, used hotel phone for OAT appointments. 2 weeks in hotel: achieved 90mg methadone therapeutic dose, reduced fentanyl use, took antiretroviral therapy more consistently, reconnected with sister and daughter, pursued volunteering opportunities. Ejected from hotel after an altercation. Started missing methadone doses, increased fentanyl use: restarted methadone.
Homeless people hospitalized with COVID-19 in Brussels	Schrooyen et al. (2021), Belgium	Cross-sectional	PEH (6% of sample). Incidence of hospitalisation=3 times higher for PEH. High but similar proportion of comorbidities present in both groups. PEH were more likely to: smoke; have AUD, neurological disease; receive treatment methadone for OST.
Creating an e-cohort of individuals with lived experience of homelessness and	Song et al. (2021), UK	Population-based retrospective e-cohort	1286 identified as PEH since 2014 in Wales, 11% of whom died between February and July 2020. 15% of deaths were registered as COVID-19 related (vs. general population-14%).

subsequent mortality in Wales, UK			
Viral suppression rates in a safety-net HIV clinic in San Francisco destabilized during COVID-19	Spinelli et al. (2020), United States	Non-randomized observational pre/post design	Shelter-in-place and telemedicine model for primary care implemented in response to COVID-19.<br>PEH were offered significantly less telehealth visits (32%) than average population (54%). PEH had fewer no-shows for appointments during shelter-in-place (April 1 to 30, 2020) than average population pre-shelter-in-place (December 1, 2019 to February 29, 2020). Viral non-suppression was higher among PEH during shelter-in-place compared to pre-shelter-in-place.
SARS-CoV-2 seroprevalence, and IgG concentration and pseudovirus neutralizing antibody titres after infection, compared by HIV status: a matched case-control observational study	Spinelli et al. (2021), United States	Matched case-control observational study	PCR testing performed for all. 16% with HIV, also PEH. 0% w/o HIV were PEH. Experiencing homelessness not associated with COVID-19 seropositivity.
Cups for COVID: rapid implementation of a harm reduction initiative to support populations experiencing homelessness during the COVID-19 pandemic	Steer et al. (2021), Canada	Mixed Methods	Biodegradable cups used to intervene against COVID-19 spread for PEH/housing instability who share beverages. 4000 cups donated (May, 2020). Overall, cups had positive impact, but limited utility for those who do not consume alcohol.<br>Cups impact: positive behaviour change (particularly among those consuming alcohol and receiving health education), cup use increased among those who previously drank alcohol, reduced COVID-19 risk, raised COVID-19 awareness, engaged clients in disease prevention measures, decreased stigma around alcohol use. Overall, staff felt cups were useful, but time restraints limited provision of health education, disposing of cups appropriately. One service noted littering of cups: 50% of cups littered, those using drugs requested resealable water bottles to prevent heat stroke/dehydration. Sites with clients in fixed locations saw reductions/no change in littering, one outreach team noted increases in littering.
First wave of COVID-19 did not reach the homeless population in Aarhus	Storgaard et al. (2020), Denmark	Cross-sectional	All COVID-19 test results returned negative among vulnerable populations (including PEH). No differences in lateral flow tests, COVID-19 symptoms, co-morbidities, and PCR tests between PEH and rest.

Mental well-being and physical activity of young people experiencing homelessness before and during COVID-19 lockdown: A longitudinal study	Thomas et al. (2021), UK	Longitudinal study	Mental wellbeing was lower among young PEH compared to general population. Females showed significantly lower levels of self-esteem. Self-esteem associated with 16-18 age group. 71%: inactive (according to physical activity levels for this age group). Wellbeing, self-esteem physical activity increased from time point 1 to 2. Wellbeing remained lower in young PEH; self-esteem remained lower for females. Higher physical activity predicted higher wellbeing.
COVID-19 Outbreak Among Three Affiliated Homeless Service Sites-King County, Washington, 2020	Tobolowsky et al. (2020), United States	Cross-sectional	SARS-CoV-2 testing offered to all at three shelters at two time points (30 March-1 April, 2020; 7-8April, 2020). Of those spending previous night at shelter, 63% tested. Overall, 18% tested positive-with more cases detected at second testing event (10% vs 16% respectively). Positive at first testing event: 80% transported to isolation housing, 20% hospitalised. Of positive cases: 89% identified through testing events, 6% through symptom screening, 6% evaluated elsewhere. 1 April: Public health-Seattle and King county (PHSKC) and Centre for Disease Control and Prevention (CDC) set up site assessments and symptom screening, isolated ill residents, reinforced infection prevention/control practices, provided face masks, advised on sheltering-in-place. 1-11 April 2020: 14 symptom screening checks for all residents of all shelters. 20% residents hospitalised, no deaths. Repeat tests offered (for those not tested/with negative results). First positive case of COVID-19, reported 2 days of cough, shortness of breath, fever, sore throat, and runny nose-remained stable and discharged to isolation housing. All shelters: had on-site indoor bathrooms/sinks/soap, served persons 50 years or over, residents could leave if back by curfew, sleeping mats spaced ≤ 3 feet apart. Shelter A: assigned individual sleeping mats. Shelter B: housed up to 110 men in two rooms, roughly half of residents transferred to shelter C to reduce overcrowding (13 March 2020), assigned individual sleeping places, became 24-hour shelter. Shelter C: housed up to 100 men in two rooms, assigned individual sleeping places, became 24-hour shelter, did not have hand sanitizer/on-site showers/facemasks for residents, residents used shelter shuttles/public transportation to shower.
Behavioral Health and Service Usage During the COVID-19 Pandemic Among Emerging Adults Currently or Recently	Tucker et al. (2020), United States	Cross-sectional	94% knew about COVID-19. 6% contracted virus. 16% knew someone contracting virus. How participants got their information: social media (71%), TV/radio (59%), news websites (60%), friends/family (52%), service providers/health professionals (45%), newspapers (42%). Worried about contracting virus: not at all (53%), a little (32%), very (8%), extremely (7%). COVID-19 protective strategies: avoided shaking hands/other physical contact (92%), wore face mask (92%), kept 6 foot distance from others (87%), cleaned hands several times per day (86%), avoided crowded places (84%), avoided contact with people at risk of having COVID-19 (81%), cancelled/postponed personal/social activities (80%), avoided sharing

Experiencing Homelessness			cigarettes/drugs (79%), quarantined self (78%), avoided public places (78%), changed school/work arrangements (67%), stockpiled food/water (58%).<br>Mental health symptoms: hopelessness (48%), anxiety (44%), loneliness (38%), sleep problems (34%), depression (36%). Increased substance use: alcohol (16%), tobacco (20%), cannabis/marijuana (28%). Harder to attain: food (54%), clean clothes/shower (33%), safe place to sleep (29%), mental health counselling (44%), case management (42%), stable housing (42%), sexually transmitted disease services (27%), substance use services (32%).
Determinants of health-related QOL among homeless individuals during the COVID-19 pandemic	van Rüth et al. (2021), Germany	Cross-sectional	Presence of chronic alcohol consumption (39%); health insurance (68%). Issues with: mobility (30%), self-care (5%), activities (21%), pain/discomfort (48%), anxiety/depression (32%). Severe issues with: self-care or activities (0%), pain/discomfort (5%), anxiety/depression (2%). Predictors of problems with: mobility-being older; self-care being older; activities-being single; pain/discomfort-N/A; anxiety/depression-no health insurance. Both QOL measures were associated with younger age. Health-related QOL was associated with higher education.
Heterogeneity in testing, diagnosis and outcome in SARS-CoV-2 infection across outbreak settings in the Greater Toronto Area, Canada: an observational study	Wang et al. (2020), Canada	Population-based prospective cohort	4% of people in shelters tested COVID-19 positive. 0.8% of positive cases died. People in shelters were 19-fold more likely to receive COVID-19 diagnosis (vs. General population).
Rapid Creation of a Multiagency Alternate Care Site for COVID-19-Positive Individuals Experiencing Homelessness	Wang et al. (2021), United States	Unspecified - Evaluation of intervention	Community fitness centre transformed to temporary medical respite shelter, providing medically monitored isolation for PEH. Clients referred from shelters/congregate settings, hospitals or inpatient units. Daily health checks. Those with emergency needs transferred to emergency departments. Laundry/food available, infection control/harm-reduction practises encouraged. Facilitated transport of clients to and from haemodialysis.<br>Telehealth visits available: primary care (48%), mental health (16%), SUD (36%). Free prescription medication. SUD treatment: on-site recovery support, initiating buprenorphine treatment, methadone delivery, naloxone training, assistance entering residential drug treatment. 88% developed relationships with care co-ordinators (assisted with applications, stable housing, primary care provider). Characteristics: diabetes (12%), heart condition (12%), HIV/AIDS (8%), other immunosuppressant condition (4%), mental health condition (88%), duration of experiencing homelessness (median=12 months), length of stay (median=7 days). Discharge following isolation: shelter (61%), friends/family (10%), SUD treatment

			programme (2%), street encampment (2%), hospitalised (2%), transferred to other isolation facility (2%), missing/unknown (14%). Demands reduced for isolation beds and programme closed in a month (end of May, 2020).
Evaluation of Hepatitis C Test and Treat interventions Targeted at Homeless Populations (Outside London) in England During the COVID-19 Pandemic	Wilkinson et al. (2021), UK	Mixed methods	Test and treated PEH for HCV while being housed. Uptake rate of 64% in residential settings. Overall, 18% HCV antibody positive: of these, 11% were HCV ribonucleic acid (RNA) positive (of these, 69% offered treatment: of these, 90% commenced treatment).<br>*Structure*-challneges: test and treat (due to restrictions); taking blood; engaging with hotel staff; attaining testing consent. Others found it provided more freedom/flexibility. HCV awareness and reducing associated stigma helped with engaging hotel staff. Partnership working (peer supporters/local authority) was a huge advantage.<br>*Process*-advertising communicated: the different routes of HCV transmission; ease and effectiveness of HCV treatment to destigmatize HCV. Some saw incentives as positive. Others believed clients needed to be self-motivated. COVID-19 prevention strategies were adhered to, although some PEH did not socially distance. Organisers solved this by testing clients one-by-one in a room.<br>*Outcomes*-Clients were happy, relieved, proud to engage with testing (it helped recovery for other comorbidities). Clients could reflect on health during lockdown. Some felt stigmatised by promotional outlets. Other health screenings carried out. Providers believed that this intervention raised awareness and may lead to future collaborations for HCV treatment/awareness/recruitment.

COVID-19 Prevalence among People Experiencing Homelessness and Homelessness Service Staff during Early Community Transmission in Atlanta, Georgia, April-May 2020	Yoon et al. (2020), United States	Cross-sectional	RT-PCR tests offered to all. Unsheltered clients offered testing at outreach events (e.g. meal provision). Positive cases informed directly or shelters notified. Positive cases in shelter were isolated in unit at shelter or isolated until transferred to isolation hotel. Positive results provided to unsheltered cases by clinic hotline/outreach team, who located the positive cases and transferred to isolation hotel.<br>73% sheltered (of these, 2%=COVID-19 positive), 27% unsheltered (of these, 0.5%=COVID-19 positive). 98% of children/adolescents experiencing homelessness were sheltered. Sheltered (61%) and unsheltered (100%) clients completed screening for medical conditions. PEH (sheltered vs. unsheltered) characteristics: 65 years old or more (5% vs. 10%); have no underlying conditions (52% vs. 48%), diabetes (11% vs. 9%), cardiovascular disease (33% vs. 34%), chronic lung disease (14% vs. 17%), chronic kidney disease (3% vs. 3%), chronic liver disease (4% vs. 6%), immunocompromising conditions (3% vs. 8%), neurological conditions (6% vs. 9%); currently smoke (49% vs. 63%); previously smoked (14% vs. 11%); never smoked (37% vs. 26%); women aged 15-44 years and pregnant (6% vs. 6%).<br>Second round of testing: all positive results from PEH not tested previously. Shelter characteristics: >50 beds (100%), >150 beds (44%), open 24/7 (89%), 44% had congregate sleeping rooms only. Shelters improved/adapted their services due to pandemic: 56% increased spacing between beds, 78% assigned isolation spaces for suspected cases, 56% stopped admitting new clients, 89% performed symptom screening for clients, all tried to enforce social distancing and infection prevention/control and increased cleaning of surfaces, placing hand sanitiser in entrances/kitchens/shared spaces. One shelter reported undetected outbreak. Proportion of persons testing positive increased from round 1 (1%) testing to round 2 (20%); attempted to reduce overcrowding; restrictions on movement not enforced, sleeping areas at least 6 feet apart (but not head-to-toe in all cases); showers encouraged but facemasks; cleaning supplies and hand sanitiser not available.

Note: PEH = persons experiencing homelessness; COVID-19 = coronavirus disease; SARS-CoV-2 = Severe Acute Respiratory Syndrome Coronavirus-2; RT-PCR = real-time polymerase chain reaction; rRT-PCR = reverse real-time polymerase chain reaction; PCR = polymerase chain reaction; HCV = hepatitis C virus; HIV = human immunodeficiency virus; RNA = ribonucleic acid; SUD = substance use disorder; CUD = cocaine use disorder; BUD = benzodiazepine use disorder; OUD = opioid use disorder; AUD = alcohol use disorder; UD = use disorder; QOL = quality of life; OAT = opioid agonist therapy; OST = opioid substitute treatment; IAR = infection attack rates; I&Q = isolation and quarantine; PrEP = pre-exposure prophylaxis; HCA = healthcare accommodation; COPD = chronic obstructive pulmonary disease; CRU = COVID-19 Recuperation Unit; ED = emergency department; IV = intravenous; *M* = mean; w/o = without; N/A = not applicable; ICU = intensive care unit; IgG = immunoglobulin G; IgM = immunoglobulin M; PHSKC = Public Health–Seattle and King County; CDC = Centers for Disease Control and Prevention; CFR = case fatality rates; RAs = residential assistants; IEP = injecting equipment provision); MAPs = managed alcohol programmes; mg = milligram; PPE = personal protective equipment; GP = general practitioner; PTSD = post-traumatic stress disorder; TV = television; GI = gastrointestinal; AIDS = acquired immunodeficiency syndrome; ETHOS = European Typology of Homelessness and housing exclusion.
